# Rapid and cost-effective identification of microorganisms from positive blood cultures using MALDI-TOF MS

**DOI:** 10.1186/s12879-025-11129-5

**Published:** 2025-05-23

**Authors:** Maryam Mostafa Ashmawy, Sahar Mohamed Khairat, Nashwa Mohamed Reda, Mona Moheyeldin Abdelhalim

**Affiliations:** Department of Clinical and Chemical Pathology, Faculty of Medicine, Cairo University, Giza, Egypt

**Keywords:** Blood culture, Rapid identification, MALDI-TOF MS, Sepsis

## Abstract

**Introduction:**

Rapid and accurate pathogen identification is critical and has a major impact on sepsis-related mortality and morbidity rates. The aim of this study is to evaluate the performance of a simple, fast, and inexpensive method for direct processing of positive blood cultures for immediate identification of microorganisms using matrix-assisted laser desorption ionization-time of flight mass spectrometry (MALDI-TOF MS).

**Methods:**

This study was performed on 125 positive blood culture bottles collected from April 2022 to September 2023. We compared using MALDI-TOF MS for identifying isolated microorganisms from positive blood culture bottles on routine culture media versus identifying microorganisms directly from positive blood culture bottles without previous isolation on routine culture media.

**Results:**

The total number of microorganisms isolated by routine subculture methods was 128, as three bottles had two types of organisms. Out of the 128 organisms isolated by routine subculture method, 97 (75.8%) organisms were identified to species level using the direct detection method from blood culture bottles by MALDI-TOF MS. 4 (3.1%) organisms were identified to genus level only, while 3 (2.3%) were wrongly identified. This direct method could not identify 24 (18.8%) organisms. Gram-negative organisms were identified with high accuracy, achieving 90.16% (55/61) at the species level and 3.28% (2/61) at the genus level. Among Gram-positive organisms, 69.1% (38/55) were identified to the species level, while 27.3% (15/55) could not be identified. Yeast identification was limited, with only 33.3% (4/12) identified to the species level, 8.3% (1/12) to the genus level, and 41.7% (5/12) could not be identified.

**Conclusion:**

This method showed great sensitivity for the identification of Gram-negative bacteria, directly from positive blood culture bottles, followed by Gram-positive bacteria, and very low sensitivity for yeasts.

## Introduction

Bloodstream infections (BSI) are a leading cause of morbidity and mortality worldwide. This is associated with prolonged use of empiric antibiotics waiting for the results, which may not be concordant with the used empiric treatment, adding burden to the antibiotic abuse and the prevalence of multidrug-resistant organisms (MDROs). Blood culture is one of the most important diagnostic methods for BSI. Processing blood culture by traditional methods takes around 48 h after the positive alarm by the automated blood culture system to report results to clinicians. Therefore, it is critically important to develop methods to rapidly and accurately identify pathogens from positive blood cultures [1]. Various in-house protocols have been developed for the direct identification of microorganisms from positive blood culture bottles. However, many of these protocols involve complex preprocessing steps or require brief subculture periods to enhance accuracy, making them time-consuming and costly. Commercial kits, while available, are often not cost-effective and do not always outperform in-house methods [2].

Matrix-assisted laser desorption ionization-time of flight mass spectrometry (MALDI-TOF MS) has emerged as a powerful tool for the rapid and accurate identification of bacteria and yeasts with high sensitivity. This technique involves the ionization of microbial proteins using a laser beam, generating unique protein profiles for each microorganism. These profiles are then compared against a reference database by the MALDI-TOF MS software to identify the microorganism [3].

This study aims to evaluate a simplified, rapid, accurate and cost-effective approach for the direct identification of bacteria from positive blood culture bottles, bypassing the need for prior isolation on routine media.

## Materials and methods

This comparative study was performed on 125 positive blood culture bottles collected from patients admitted to Cairo University specialized pediatric hospital between April 2022 and September 2023.

The method was developed based on the method used by ***Yuan et al.*** with some modifications [1]. In this study, we only centrifuged the sample once. This decreased processing time by at least 10 min.

### Processing of blood culture

The blood culture bottles were incubated in one of the automated aerobic blood culture incubators BD BACTEC™ FX40 (Becton Dickinson, USA) or BacT/ALERT™ System (bioMérieux, France) for up to 5 days or until positive results were obtained. Direct Gram-stained film was prepared from blood culture bottles flagged as positive by the automated system and examined under the microscope to see whether they contained one or multiple organisms.

Inclusion criteria: Only samples flagged as positive by the automated aerobic blood culture system that showed a single organism in direct Gram-stained film were included.

Exclusion criteria: Negative blood cultures and blood cultures that showed no organism or more than one organism in direct Gram-stained film.

### Identification of organisms by the routine subculture method

Blood from positive blood culture bottles was sub-cultured on blood, MacConkey, and chocolate agar. Agar plates were incubated at 36 ± 1 °C for 18–24 h. Following incubation, isolated colonies were spotted on a MALDI-TOF MS target plate and subjected to MALDI-TOF MS analysis. We used VITEK^®^ MS V3.2 Knowledge Base for identification of isolated microorganisms.

### Identification of microorganisms directly from positive blood culture bottles

1. 4.0 mL of blood was taken from positive blood culture bottle fulfilling determined inclusion criteria of either BD BACTEC™ FX40 (Becton Dickinson, USA) or BacT/ALERT™ System (bioMérieux, France) and transferred to a tube containing plasma separation gel, which was centrifuged at 3000 g for 10 min.
2. After discarding the supernatant, 1.0 mL of deionized water was added to resuspend the precipitate.
3. Next, 1 µL of the suspension was spotted on a MALDI-TOF MS target plate and subjected to MALDI-TOF MS analysis. Triplicate spots were generated for each sample.

### MALDI-TOF MS analysis

**Fig. 1:**
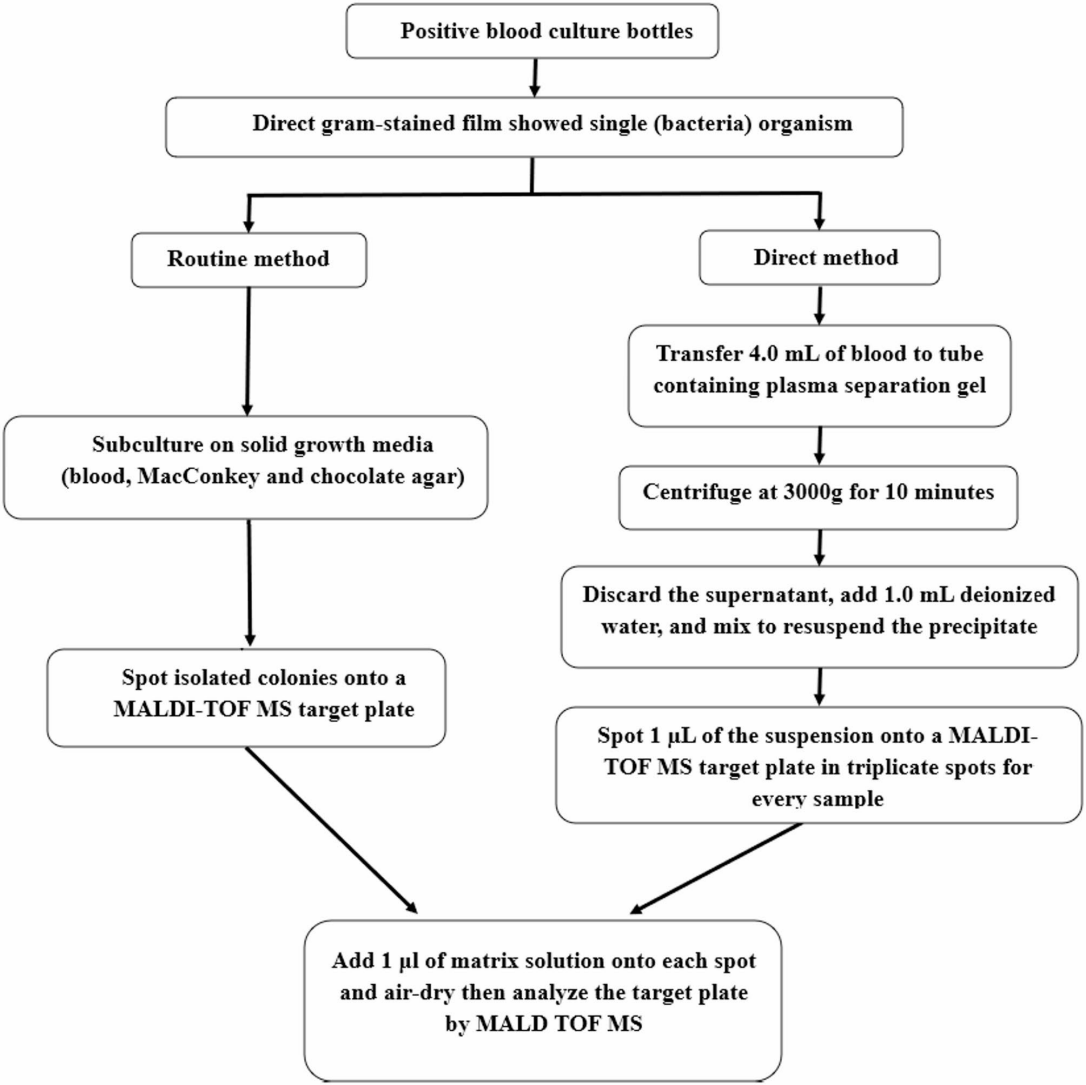
Diagram showing the plan of our work for bacteria

1. After adding the microorganism to the MALDI-TOF MS target plate, we added the matrix solution. There was a different technique for bacteria and yeast:

A) In the case of bacteria (Fig. 1**)**:

- 1 µl of alpha-cyano-4-hydroxycinnamic acid (CHCA) matrix solution was placed onto each spot and left to completely dry within 30 min.

**Fig. 2:**
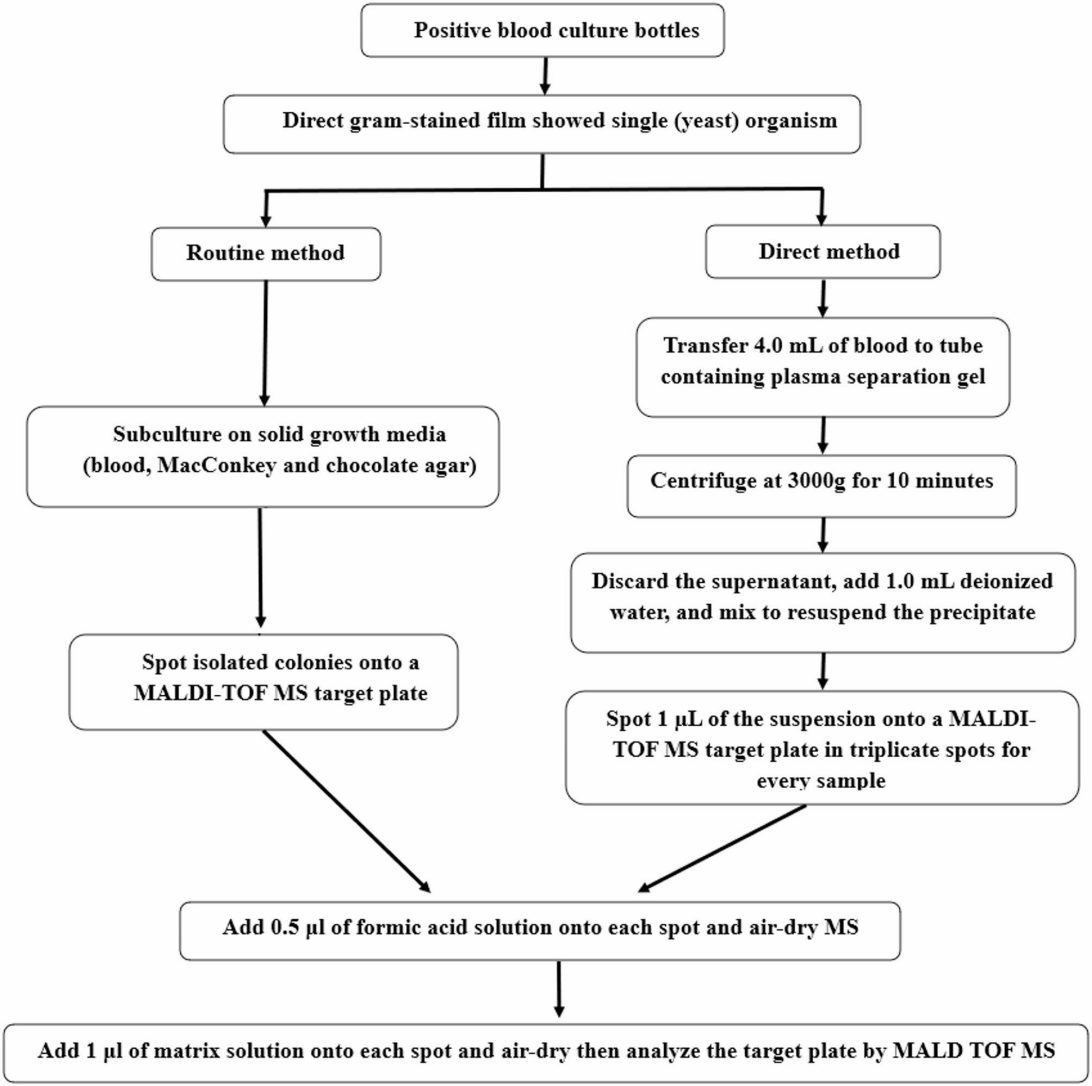
Diagram showing the plan of our work for yeast

B) In the case of yeast (Fig. 2**)**:

- 0.5 µL of formic acid (FA) was added to the spot using a pipette and left to allow evaporation of the FA before adding the matrix.
- 1 µl of CHCA matrix solution was placed onto each spot and left to completely dry within 30 min.

2) The target plate was analyzed by the Vitek^®^ MS V3.2 (bioMérieux system, France).

QC of Vitek-MS was performed with each run using *E-coli* ATCC 8739 according to manufacturers’ instructions. The *E. coli* ATCC^®^ 8739™ strain was incubated for 18 to 24 h at 35 °C ± 2 °C on Columbia agar + 5% sheep blood. The negative control was performed using 1 µl of VITEK^®^ MS-CHCA matrix which gave no ID.

### Interpretation

The identification of microorganisms by MALDI-TOF MS was performed using a software application that compares the spectral profile of the unknown organism with a reference database containing 1316 taxa. The analysis provides the best identification match along with a confidence score ranging from 0 to 99.9%. A confidence score of 95 to 99.9% was considered high at the species level, while a score of 90 to 94% was considered high at the genus level. For confidence scores between 50 and 94%, the result was recorded as ‘genus-level’ if the match included 2–4 organisms within the same genus. If the organisms belonged to different genera, no valid identification was recorded.

### Statistical analysis

Sample size calculation was based on the sensitivity of a rapid diagnostic method for identification of micro-organisms directly from positive blood culture bottles using MALDI-TOF MS. Prior data indicated that the sensitivity of the rapid diagnostic method in identification of micro-organisms directly from positive blood culture ranged from 67.69 to 99% [3, 14] with an average of 83.4% ± 16%. We needed to study at least 72 positive blood cultures to be able to reject the null hypothesis with 80% power setting the type I error probability to 0.05, assuming that this was the true population sensitivity. Calculations were done using Flahault et al., Eq. (2005) [4].

Categorical data was described using the frequencies (number of organisms) and percentages for each category as well as the total sample. The frequency was the number of organisms recorded in every category. The percentage was calculated by dividing the number of organisms in each category by the total number of samples multiplied by 100. The sensitivity of the rapid direct method in the identification of microorganisms in positive blood culture was calculated by comparing the direct MALDI-TOF method to the subculture method.

## Results

The total number of positive blood culture bottles was 125 (108 BD BACTEC™ FX40 and 17 BacT/ALERT™), from which 128 micro-organisms were isolated by the subculture method as three bottles had two types of organisms that could not be differentiated by the Gram staining. Two showed Gram-positive bacteria and one showed Gram-negative bacteria of different types (Table 1) (Table 2).

**Table 1:** The classification of micro-organisms according to direct gram staining and routine subculture

Category	By Gram staining		By routine subculture	
Gram-positive	**53**	**42.4%**	**55**	**42.97%**
Gram-negative	**60**	**48.0%**	**61**	**47.66%**
Yeast	**12**	**9.6%**	**12**	**9.37%**
Total	**125**	**100%**	**128**	**100%**

**Table 2:** Identification of organisms from bottles that contained more than one organism

Organisms identified by Gram stain	Organisms identified by routine subculture	Organisms identified by direct method
Gram positive cocci	*Staph hominis + Staph epidermidis*	*Staph hominis*
Gram positive cocci	*Lactococcus lactis + Staph hominis*	*Lactococcus lactis*
Gram negative bacilli	*klebsiella pneumoniae + Enterobacter cloacae50%/asburiae50%*	*klebsiella pneumoniae*

### Direct detection by MALDI-TOF MS

**Fig. 3:**
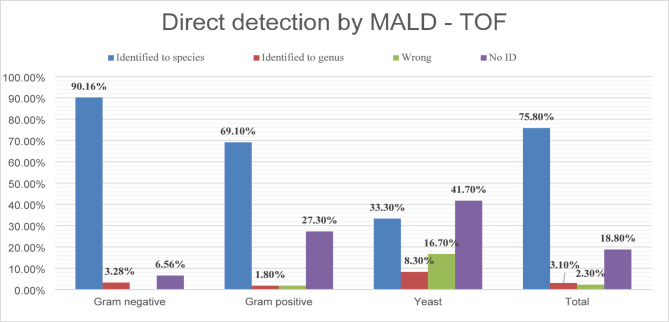
The percentage of different categories of microorganisms correctly identified directly from blood culture bottles by the MALDI-TOF MS method

Out of the 128 organisms isolated by the subculture method, MALDI-TOF MS directly identified 97 (75.8%) microorganisms at the species level, four (3.1%) at the genus level, and three (2.3%) were misidentified. Twenty-four (18.8%) microorganisms could not be identified (Fig. 3).

The shorter the time to positivity of the automated blood culture bottles, the more accurate the identification of microorganisms by MALDI-TOF MS directly from positive blood culture bottles with significant P-values as shown in Table 3.

**Table 3:** The relation between time to positivity of the blood culture bottle and accuracy of identification to genus and species level by MALDI-TOF MS, directly from positive blood culture bottles

Level of identification	Correct identification		Wrong or No ID		*P* value *
	Median time in hours	Interquartile range	Median time in hours	Interquartile range	
Genus identification	11.99	9.8–17.6	19.1	13.4–24.8	0.002
Species identification	11.99	9.8–17.3	19.1	13.05–24.6	0.004

*: Mann-Whitney test, P value < 0.05 ◊ Statistically significant difference, P value > 0.05 ◊ No statistically significant difference, Correct identification by the direct method, Wrong or No ID by the direct method

### Identification of Gram-negative bacteria directly from positive blood culture bottles by the MALDI-TOF MS method (Table 4)

**Table 4:** Distribution of different Gram-negative bacteria correctly identified directly from positive blood culture bottles by MALDI-TOF MS

Gram-negative bacteria	Total*	Identified to species		Identified to genus		No Id	
		No.	%	No.	%	No.	%
*Klebsiella pneumoniae*	**26**	**25**	**96.2%**	**0**	**0**	**1**	**3.8%**
*Klebsiella oxytoca*	**2**	**2**	**100%**	**0**	**0**	**0**	**0**
*Pseudomonas aeruginosa*	**10**	**10**	**100%**	**0**	**0**	**0**	**0**
*Pseudomonas putida*	**2**	**2**	**100%**	**0**	**0**	**0**	**0**
*E. coli*	**5**	**4**	**80%**	**0**	**0**	**1**	**20%**
*Acinetobacter baumannii*	**4**	**4**	**100%**	**0**	**0**	**0**	**0**
*Citrobacter Freundii*	**3**	**1**	**33.3%**	**2**	**66.7%**	**0**	**0**
*Serratia marcescens*	**2**	**2**	**100%**	**0**	**0**	**0**	**0**
*Proteus mirabilis*	**2**	**2**	**100%**	**0**	**0**	**0**	**0**
*Morganella morganii*	**1**	**1**	**100%**	**0**	**0**	**0**	**0**
*Haemophilus influenzae*	**1**	**1**	**100%**	**0**	**0**	**0**	**0**
*Providentia stuartii*	**1**	**1**	**100%**	**0**	**0**	**0**	**0**
*Burkholderia stabitis*	**1**	**0**	**0**	**0**	**0**	**1**	**100%**
*Enterobacter SPP*	**1**	**0**	**0**	**0**	**0**	**1**	**100%**
Total	**61**	**55**	**90.16%**	**2**	**3.28%**	**4**	**6.57%**

*Total = total number of organisms identified by MALDI-TOF MS from isolated colonies, identified to species by the direct method, Identified to genus by the direct method, No Id by the direct method

### Identification of gram-positive bacteria directly from positive blood culture bottles by the MALDI-TOF MS method

One isolate of *Staphylococcus hominis* was misidentified as *Bacillus subtilis* and one isolate of *Staphylococcus epidermidis* was identified as *Staphylococcus aureus* compared by routine identification of isolated colonies by MALDI-TOF MS (Table 5).

**Table 5:** Distribution of different Gram-positive bacteria correctly identified directly from positive blood culture bottles by MALDI-TOF MS

Gram-positive organism	Total*	Identified to species		Identified to genus		Wrong ID		No Id	
		No.	%	No.	%	No.	%	No.	%
*Staphylococcus hominis*	**17**	**12**	**70.6%**	**0**	**0**	**1**	**5.9%**	**4**	**23.5%**
*Staphylococcus epidermidis*	**11**	**4**	**36%**	**1**	**9%**	**0**	**0**	**6**	**55%**
*Staphylococcus aureus*	**10**	**10**	**100%**	**0**	**0**	**0**	**0**	**0**	**0**
*Staphylococcus haemolyticus*	**4**	**3**	**75%**	**0**	**0**	**0**	**0**	**1**	**25%**
*Streptococcus mitis/oralis*	**3**	**3**	**100%**	**0**	**0**	**0**	**0**	**0**	**0**
*Streptococcus SPP*	**2**	**1**	**50%**	**0**	**0**	**0**	**0**	**1**	**50%**
*Enterococcus feacalis*	**2**	**1**	**50%**	**0**	**0**	**0**	**0**	**1**	**50%**
*Lactococcus lactis*	**1**	**1**	**100%**	**0**	**0**	**0**	**0**	**0**	**0**
*Kocuria palustris*	**1**	**1**	**100%**	**0**	**0**	**0**	**0**	**0**	**0**
*Bacillus cereus*	**1**	**1**	**100%**	**0**	**0**	**0**	**0**	**0**	**0**
*Aerococcus*	**1**	**1**	**100%**	**0**	**0**	**0**	**0**	**0**	**0**
*Bacillus licheniformis*	**1**	**0**	**0**	**0**	**0**	**0**	**0**	**1**	**100%**
*Streptococcus salivaris*	**1**	**0**	**0**	**0**	**0**	**0**	**0**	**1**	**100%**
Total	**55**	**38**	**69.1%**	**1**	**1.8%**	**1**	**1.8%**	**15**	**27.3%**

*Total = total number of organisms identified by MALDI-TOF MS from isolated colonies, Identified to species by the direct method, Identified to genus by the direct method, Wrong ID by the direct method, No Id by the direct method

### Identification of yeasts directly from positive blood culture bottles by MALDI-TOF MS method

Two isolates were wrongly identified. One *Candida albicans* and one *Candida tropicalis* were both identified as *Aspergillus terreus* complex compared by routine identification of isolated colonies by MALDI-TOF MS (Table 6).

**Table 6:** Percentage of different yeasts correctly identified directly from positive blood culture bottles by MALDI-TOF MS

Yeasts	Total*	Identified to species		Identified to genus		Wrong ID		No ID	
		No.	%	No.	%	No.	%	No.	%
*Candida albicans*	**5**	**1**	**20%**	**0**	**0**	**1**	**20%**	**3**	**60%**
*Candida parapsilosis*	**3**	**2**	**66.7%**	**0**	**0**	**0**	**0**	**1**	**33.3%**
*Candida glabrata*	**1**	**1**	**100%**	**0**	**0**	**0**	**0**	**0**	**0**
*Candida dubliniensis*	**1**	**0**	**0**	**1**	**100%**	**0**	**0**	**0**	**0**
*Candida pelliculosa*	**1**	**0**	**0**	**0**	**0**	**0**	**0**	**1**	**100%**
*Candida tropicalis*	**1**	**0**	**0**	**0**	**0**	**1**	**100%**	**0**	**0**
Total	**12**	**4**	**33.3%**	**1**	**8.3%**	**2**	**16.7%**	**5**	**41.7%**

*Total = total number of organisms identified by MALDI-TOF MS from isolated colonies, Identified to species by the direct method, Identified to genus by the direct method, Wrong ID by the direct method, No Id by the direct method

## Discussion

Directly identifying organisms from positive blood cultures using MALDI-TOF MS is considered a great achievement in the microbiological diagnosis of bloodstream infections. It necessitates separating the organism from other potentially disturbing signals, such as human blood cells, culture media, and other debris found in the blood culture broth [5].

Several bacterial extraction techniques are available, such as serum separator tube [1, 3, 6], centrifugation and filtration techniques [7, 8], differential velocity centrifugation [5], Sepsityper method used by [5, 8], and the Saponin method [9].

Centrifugation using the serum separator gel tubes method was used in this study. This approach saved a significant amount of time by identifying organisms to the species level from positive blood cultures within an hour. This procedure was simple, easy to apply, and cost-effective. No specialized kits or chemical reagents were required.

In this study, three polymicrobial cases were included, although their direct Gram-stained film showed only one organism. By the direct method, only one organism was identified in these polymicrobial cases. This was also reported by ***Tadros and Petrich*** and ***Almuhayawi et al.*** [10, 11]. Identifying each microorganism in polymicrobial cultures has been one of the direct method’s limitations. An abnormal protein spectrum is generated during the MALDI-TOF MS identification process of polymicrobial samples, leading to a mixture of several profiles. In these situations, the microorganisms cannot be identified by MALDI-TOF MS from its database [12]. Identifying and excluding polymicrobial culture just from the Gram staining proved to be challenging, as a result, there is still a possibility that some components of polymicrobial cultures may be missed by rapid identification techniques [8]. Polymicrobial sepsis is not common and, in most cases, it is considered contamination, however, identification of polymicrobial infection is important to be confirmed by another set of blood cultures.

Beyond the known challenges of identifying polymicrobial bloodstream infections, several additional limitations should be acknowledged when interpreting the findings of this study. The relatively modest sample size, especially for certain organism groups such as yeasts, may have limited the statistical strength of subgroup analyses. Moreover, while the method proved simple and cost-effective, the manual nature of several processing steps including centrifugation, pellet resuspension, and matrix application introduces a degree of operator variability. This could potentially impact reproducibility across laboratories with different levels of experience or resource availability. Testing each sample in triplicate may help decrease such potential error. Future studies involving larger, multi-center datasets and standardized protocols are recommended to validate these findings and improve robustness.

Similar limitations have been reported in previous studies evaluating direct identification methods from blood culture bottles. For instance, ***Samaranayake et al. and Huang et al.*** highlighted the challenge of reproducibility due to operator-dependent steps such as pellet handling and matrix application, which may vary across laboratories and influence identification outcomes [2, 9]. Additionally, ***Ceballos-Garzón et al.*** emphasized that smaller sample sizes, particularly for less frequent pathogens like yeasts, limit the ability to draw broad conclusions and often underestimate the variability in performance [21]. Moreover, ***Kayin et al. and Ponderand et al.*** demonstrated that even well-structured in-house methods may show reduced sensitivity in real-world settings unless standardized across multiple operators and institutions [5, 20]. These findings reinforce the importance of larger, multicenter studies with more standardized workflows to overcome these inherent limitations and improve the generalizability of direct MALDI-TOF protocols.

The relation between time to positivity (TTP) of the automated blood culture bottles and the ability of the direct method to correctly identify the organisms was significant. Median Time to positivity was significantly shorter among correctly identified bottles. This was in agreement with ***Fang et al.***, who also found that the rapid protocols exhibited better performance with a shorter time to positivity of blood cultures (< 19 h) [13]. The idea is that a shorter TTP may be associated with a greater bacterial load, which raises mortality. This was supported by the majority of previous studies which showed a strong association between short TTP and mortality [14].

Gram-negative bacteria showed the highest sensitivity in this study using the direct method (90.16%). Followed by Gram-positive bacteria (69.1%), while yeasts showed the lowest sensitivity (33.3%). These findings were consistent with ***Yuan et al.***,*** Samaranayake et al.***, ***Kayin et al.***,*** Yonetani et al.***, and ***Park and Kim*** showing that Gram-negative bacteria could be identified by MS more precisely than Gram-positive bacteria [1, 2, 5, 15, 16].

Although the exact cause remains unclear, several factors have been proposed to explain why Gram-positive bacteria are generally more challenging to identify than Gram-negative bacteria. The efficiency of protein extraction can be hindered by the relatively thick peptidoglycan layer in the cell wall of Gram-positive bacteria, which impedes efficient protein extraction, a critical step in MALDI-TOF MS analysis. Several studies have shown that additional formic acid treatment or mechanical disruption are often needed to improve lysis and protein release for these organisms [1, 5, 9]. Additionally, a higher bacterial load may be required to achieve adequate protein yield for analysis [15, 20]. Certain Gram-positive species exhibit slow growth rates, which can result in insufficient biomass for effective extraction [2]. Furthermore, due to the high degree of structural similarity among some Gram-positive cocci, there is an increased risk of misidentification [15, 17]. Therefore, when Gram-positive cocci are detected in blood culture bottles, results obtained through direct identification methods should be interpreted with caution.

Identifying Yeast using MALDI-TOF MS is challenging. Their thick cell wall may be the cause. It is recommended to perform preliminary preparation steps and more complicated processing to rupture the Yeast cell wall and release intracellular proteins to identify Yeasts more precisely and accurately [2, 3].

In Gram-negative bacteria, the direct identification method showed 100% sensitivity for *Pseudomonas aeruginosa* and *Acinetobacter baumannii*, 96.2% sensitivity for *Klebsiella pneumoniae*, and 80% for *E. coli*. *Citrobacter Freundii* showed 33.3% sensitivity to the species level and 100% to the genus level. These results were consistent with ***Barth et al.***,*** Riederer et al.***, and ***Cruz et al.***, which showed high sensitivity to common Gram-negative bacteria such as *Pseudomonas aeruginosa* (94.6-100%), Acinetobacter *baumannii* (85.70-100%), *E. coli* (96.7-100%), and *Klebsiella pneumoniae* (92-97.3%). The lower sensitivity to *E. coli* in our study could be explained by the low total number of *E. coli* cases as it was isolated from five samples only [6, 18, 19].

In Gram-positive bacteria, *Staphylococcus aureus* showed 100% sensitivity using the direct method of identification, while CoNS showed 59.4% sensitivity. *Streptococci* showed 83.3% sensitivity. ***Samaranayake et al.***,*** Huang et al.***, and ***Ponderand et al.*** also showed high sensitivity to *Staphylococcus aureus* (81.1-100%) [3, 9, 20],. These studies showed much lower sensitivity to *Streptococci* (50-60.6%), which could be due to the high similarity in the protein profiles between *Streptococci* species. These studies showed higher sensitivity to CoNS (81.93-93.65%).

The number of Yeasts was very low (12 isolates), which limited our ability to evaluate this direct method in identifying yeasts. *Candida parapsilosis* showed the highest sensitivity at 66.7%. *Candida albicans* was correctly identified in one out of five cases (20%). Only one *Candida glabrata* was encountered and correctly identified. ***Huang et al.*** showed similar low sensitivity to *Candida albicans* (20%) and lower sensitivity to *Candida parapsilosis* (33.3%) which suggested that their in-house method needs further improvement [9]. In ***Yuan et al.****Candida glabrata* and *Candida parapsilosis* were identified accurately in 100% of cases, followed by *Candida albicans* and *Candida tropicalis*, each showed 80% sensitivity [1]. The method used by ***Samaranayake et al.*** and ***Ceballos-Garzón et al.*** failed to identify Yeasts directly from positive blood culture [2, 21].

Despite the advantages of this method, some limitations were noted, particularly the lower identification accuracy for yeasts. And the inability to be used in samples containing more than one organism. Further studies on a wider scale with larger sample sizes and additional optimization to detect yeasts and uncommon organisms are recommended. New research to develop a technique to process blood cultures that showed more than one organism in direct Gram-stained film is also recommended.

## Conclusion

Matrix-Assisted Laser Desorption Ionization–Time of Flight Mass Spectrometry (MALDI-TOF MS) enables the rapid and cost-effective identification of common Gram-negative and Gram-positive microorganisms directly from positive blood culture bottles. When integrated with the institution’s antibiogram data, this approach can facilitate earlier initiation of targeted antimicrobial therapy, thereby potentially improving clinical decision making. However, the method demonstrates limited sensitivity in the detection of Yeast species.

### Limitations

This method demonstrated limited efficacy in the identification of Yeast species and was not applicable to polymicrobial samples. Furthermore, the small sample size and the restriction of sample collection to a single hospital constrained the generalizability of the findings and hindered comprehensive evaluation of the method’s performance in identifying rare or uncommon organisms. In addition, most blood culture bottles in our study were processed using the BD BACTEC™ FX40 system, with only a small proportion (17/125) from the BacT/ALERT™ system. This imbalance limited our ability to perform a meaningful comparison of performance across different automated systems.

## Data Availability

The datasets generated during and analyzed during the current study are available from the corresponding author on reasonable request.

## Abbreviations

MALDI-TOF MS: Matrix-assisted laser desorption ionization-time of flight mass spectrometry
BSI: Bloodstream infections
MDROs: Multidrug-resistant organisms
